# Novel QTL Conferring Phosphorus Acquisition and Utilization Efficiencies in Barley

**DOI:** 10.3389/fgene.2020.580452

**Published:** 2020-09-04

**Authors:** Shangqing Gao, Jiaqi Xia, Shu Yuan, Youjie Shen, Xinting Zhong, Senfeng Zhang, Yuhang Li, Deyi Hu, Jian Zeng, Ting Lan, Yaxi Liu, Guangdeng Chen

**Affiliations:** ^1^College of Resources, Sichuan Agricultural University, Chengdu, China; ^2^Triticeae Research Institute, Sichuan Agricultural University, Chengdu, China

**Keywords:** barley, phosphorus deficiency, phosphorus acquisition, phosphorus utilization, quantitative trait loci

## Abstract

Phosphorus (P) deficiency in agricultural soil is a major constraint for crop production and increasing P acquisition efficiency (PAE) of plants is considered as one of the most cost-effective solutions for yield increase. The objective of this study was to detect quantitative trait loci (QTL) controlling (PAE) and P utilization efficiency (PUE) in barley under applied (+P) and non-applied P (−P) conditions. Based on the analysis of a recombinant inbred lines (RILs) population derived from a cross between a malting barley variety and a wild barley accession, 17 QTL controlling PAE, PUE and yield traits were detected. The phenotypic variation explained by each of these QTL ranges from 11.0 to 24.7%. Significant correlation was detected between most of P-related traits and yield traits. Five QTL clusters were identified on four different chromosomes (1H, 3H, 5H, and 7H). Two of the QTL clusters, located on chromosome 1H (for GPUP/PUP) and 7H (for SPUE/SPC), respectively, are novel. Fourteen genes located in the interval harboring the major QTL were identified as candidates associated with P efficiency. The stable QTL for PAE, PUE and yield-related traits could be important for breeding P-efficient barley varieties.

## Introduction

Phosphorus (P) is one of the most important mineral nutrient elements for plant development and it plays an irreplaceable role in agricultural productions (Su et al., 2006; Wang and Yan, 2010; Noack et al., 2014). Although agronomic inputs of P fertilizer and manure collectively exceeded P removal by harvested crops at the global scale, P deficits covered almost 30% of the global cropland area (Macdonald et al., 2011). The application of P fertilizers is one of the most effective methods to alleviate soil P deficiency (Shen et al., 2011). However, most of the applied P may be immobilized by calcium (Ca) and magnesium (Mg) in alkaline soils or by ferrum (Fe) and aluminum (Al) in acid soils (Holford, 1997; Yang et al., 2011). Thus only 10–20% P could be absorbed in the year of application (Yang et al., 2011). The mineral phosphate is non-renewable (Sattari et al., 2012) and the un-absorbed P will run-off into surface water to cause eutrophication (Carpenter, 2008). It is widely believed that developing cultivars with high-efficiency P acquisition and utilization in P-deficient soils is one of the most economical and sustainable solutions in crop breeding programs worldwide (Yan et al., 2006; Liang et al., 2010).

It has been reported that P efficiency in crops was affected by a number of quantitative trait loci (QTL) (Yang et al., 2011). Based on mechanisms developed by plants to acquire and utilize P from the soils (Yang et al., 2011), the mapped QTL could be classified into two major types: for P acquisition efficiency (PAE) and for P utilization efficiency (PUE) (Yang et al., 2011). QTL for P efficiency have been identified in several crops including common wheat (*Triticum aestivum*) (Su et al., 2006), maize (*Zea mays*) (Cai et al., 2012), and rice (*Oryza sativa*) (Nishida et al., 2018). In wheat, a large number of P efficiency-related QTL have been detected. For example, Su et al. (2006) detected several QTL on seven different chromosomes (3B, 4B, 5A, 5D, 6A, 6B, and 7A, respectively) for PUE under P deficient and sufficient conditions. Four important QTL clusters controlling PAE and PUE were found at both seedling and mature stages of plant development (Yuan et al., 2017) (Six QTL for PAE were co-located with the QTL for zinc concentration or content in wheat grains (Shi et al., 2008). Therefore, genome-wide scanning for QTL controlling PAE and PUE could be an important work in crop breeding programs.

Barley (*Hordeum vulgare* L.) is the fourth largest cereal crop worldwide, and it is widely used as animal feed and in brewing industry (Schulte et al., 2009). Numerous QTL or genes for important traits of barley have been mapped, fine mapped or even cloned, and they showed great potential in MAS (Peighambari et al., 2005; Tavakol et al., 2015; Li et al., 2016). However, only a limited number of QTL for P efficiency have been reported (Gong et al., 2016; Guo et al., 2017), especially from mature plants. In the present study, the whole genome linkage map of a recombinant inbred lines (RIL) population derived from the cross between a wild barley accession and a cultivar was used to detect QTL for PAE, PUE and yield at maturity stage under both P applied and non-applied soil conditions. Overall, the objective of our study was to focus on excavating the major and stable QTL or QTL clusters that could provide available information for the barley breeding programs.

## Materials and Methods

### Plant Materials

An RIL population consisting of 128 F_7:9_ lines derived from a cross between Baudin, a high yielding malting variety adjust to a longer season, higher rainfall cropping region and parts of the medium rainfall cropping region of Western Australia and a wild barley (*H. spontaneum*) accession, CN4027 was used in this study.

### Experimental Design

The calcareous alluvial soil used in pot trials was collected from Shuangbai village, Dujiangyan city in Sichuan, China. The physicochemical properties of the soil were shown in Table 1. Two pot trials were carried out (one from November 2016 to June 2017 and the other from November 2017 to June 2018) in the rainproof screenhouses of Sichuan Agricultural University. Each of the trials consisted of two treatments [P deficiency (−P, without P application) and P sufficiency (+P, 30 mg phytate P was applied per kilogram soil)] with three replications. Split plot arrangement was used in these trials. Twelve kg air-dried soil with 1.5 g N and 1.5 g K was crushed and uniformly mixed.

**TABLE 1:** Physicochemical property of the experimental soil.

**Classification**	**Values**	**Units**
Soil pH	6.89	–
Organic content	15.8	g kg^–1^
Total nitrogen (N)	0.4	g kg^–1^
Alkali-hydrolyzable N	44.68	mg kg^–1^
Available P	5.14	mg kg^–1^
Rapidly available kalium (K)	23.69	mg kg^–1^
Ca_2_−P	7.25	mg kg^–1^
Ca_8_−P	3.97	mg kg^–1^
Ca_10_−P	230.67	mg kg^–1^
Al−P	16.2	mg kg^–1^
Fe−P	76.85	mg kg^–1^
Organic−P	100.54	mg kg^–1^
Active phytate P	2.25	mg kg^–1^
Secondary active phytate P	145.12	mg kg^–1^
Secondary stable phytate P	39.75	mg kg^–1^
High stable phytate P	8.08	mg kg^–1^

For each replication, 10 uniformly sized seeds of each of RILs as well as the parents were surface-sterilized by soaking in a 10% solution of hydroperoxide (H_2_O_2_) for 30 min followed by washing in deionized water. The disinfected seeds were placed in a chamber with constant temperature humidity (20°C, 60% humidity) for germination. Five germinated seeds were planted in each of the pots. Seedlings were thinned at 3-leaf stage and three seedlings were retained in each pot and used for further experiments.

### Phenotypic Evaluation

Data for grain yield (GY), straw yield (SY), and dry matter (DM) were collected at maturity (Table 2). Harvested kernels and straws were placed in an over at 105°C for 30 min and then dried at 75°C until constant weights were reached. The dried materials were weighed and grounded to powder for determining phosphorus content with the H_2_SO_4_-H_2_O_2_-molybdenum antimony colorimetric method (Guo et al., 2017). The evaluated traits were listed in Table 2.

**TABLE 2:** The investigated traits and the measurements in this study.

**Type**	**Trait**	**Abbreviation**	**Method**	**Unit**
PAE	Grain P concentration	GPC	Measure	g plant^–1^GY
	Straw P concentration	SPC	Measure	g plant^–1^SY
	Plant P concentration	PC	PUP/DM	g plant^–1^DM
	Grain P uptake	GPUP	GY × GPC	g plant^–1^
	Straw P uptake	SPUP	SY × SPC	g plant^–1^
	Plant P uptake	PUP	GPUP+SPUP	g plant^–1^
PUE	Grain P utilization efficiency	GPUE	GY/PUP	g GY g^–1^P
	Straw P utilization efficiency	SPUE	SY/PUP	g SY g^–1^P
	Plant P utilization efficiency	PUE	DM/PUP	g DW g^–1^P
Yield trait	Grain yield	GY	Measure	g plant^–1^
	Straw yield	SY	Measure	g plant^–1^
	Dry matter	DM	GY + SY	g plant^–1^

*PAE, P acquisition efficiency; PUE, P use efficiency.*

### QTL Mapping

Means of the traits for each RIL from the three replications were used to detect QTL. The genetic linkage map obtained from this population in an earlier study (Guo et al., 2017) was used for QTL mapping. Linkage analysis was carried out using the computer package JoinMap^®^4.0 (Van Ooijen, 2006). Segregation ratios of assessed markers were tested by Chi-square goodness-of-fit to a 1:1 ratio at the significance level of *p* = 0.01. LOD thresholds from 3 to 10 were tested and an optimum threshold was obtained. The Kosambi mapping function was used to convert recombination ratios to map distances. MapQTL^®^ 5.0 (Van Ooijen, 2004) was used for QTL analysis. QTL were named following recommendations from the International Rules of Genetic Nomenclature^1^.

### Identification of Candidate Genes

To identify candidate genes for P-related loci, sequences of DArT markers linked closely to QTL were selected from the DArT genotyping provided by the Triticarte Pty. Ltd^2^. The database Ensembl Plants^3^ was exploited to determine the physical positions and contigs of the P-related loci. Candidate genes were then further retrieved using physical position and contigs by database BARLEX searches (the Barley Draft Genome Explorer^4^). Orthologous genes for the candidate genes in other cereals and *Arabidopsis* were obtained from the PGSB database^5^.

## Results

### Phenotypic Variation of Assessed Traits

Phenotype values for each trait were significantly influenced by the application of P. GY, SY, and DM of the parents at −P were significantly lower than those under +P (Table 3). At the same P condition, significant differences between parents were detected for GY, SY, and DM. The cultivated barley Baudin exhibited higher values for each of the traits compared with those for the wild barley genotype CN4027 (Table 3). The coefficient of variation (*CV*) for the yield-related traits between the two treatments ranged from 28.30 to 52.99% (Table 3). Transgressive segregation in the RIL population was observed for all three traits (Table 3). Continuous variations with approximately normal distributions were detected for these traits.

**TABLE 3:** Variations of evaluated traits for the RIL population and their parents at maturity.

**Types**	**Traits**	**Treatments**	**Trial 1**						**Trial 2**					
			**Parents**		**RILs**				**Parents**		**RILs**			
			**Baudin**	**CN4027**	**Mean + SD**	**Min**	**Max**	***CV*%**	**Baudin**	**CN4027**	**Mean + SD**	**Min**	**Max**	***CV*%**
PAE	GPC	−P	2.108	1.620	2.835 ± 1.025	0.902	5.385	36.15	2.361	1.814	3.050 ± 1.182	0.876	6.031	38.75
		+P	3.831	2.890	3.188 ± 1.019	1.459	6.104	31.96	3.534	3.054	3.308 ± 1.052	1.337	6.466	31.80
	SPC	−P	0.981	0.467	0.963 ± 0.296	0.568	1.756	30.73	1.100	0.610	0.901 ± 0.348	0.412	1.832	38.62
		+P	1.521	0.820	1.145 ± 0.396	0.614	3.056	34.58	1.302	1.000	1.190 ± 0.430	0.529	2.678	36.13
	PC	−P	1.571	1.171	1.951 ± 0.671	0.866	3.706	34.39	1.747	1.211	2.033 ± 0.836	0.850	4.137	41.12
		+P	2.697	1.991	2.201 ± 0.839	1.069	4.775	38.11	2.443	2.166	2.280 ± 0.895	1.026	4.919	39.25
	GPUP	−P	8.527	5.176	12.199 ± 4.821	2.813	28.472	39.51	8.786	5.333	12.464 ± 5.274	2.937	32.129	42.31
		+P	18.508	11.387	16.341 ± 10.559	1.478	73.363	64.61	19.292	13.597	18.308 ± 11.451	5.119	78.005	62.54
	SPUP	−P	3.610	0.951	3.677 ± 1.119	1.845	6.535	30.43	3.886	1.803	3.482 ± 1.483	1.719	7.156	42.59
		+P	7.086	2.481	5.136 ± 2.349	1.713	10.805	45.73	6.794	3.389	5.911 ± 2.720	1.777	11.709	46.01
	PUP	−P	12.137	6.127	15.877 ± 5.571	6.037	30.826	35.08	12.672	7.136	16.093 ± 6.763	6.608	34.861	42.02
		+P	25.594	13.868	21.493 ± 11.306	4.700	79.112	52.60	26.086	16.986	24.273 ± 12.558	8.031	85.152	51.73
PUE	GPUE	−P	0.474	0.617	0.400 ± 0.189	0.186	1.109	47.25	0.424	0.551	0.381 ± 0.193	0.166	1.008	50.65
		+P	0.261	0.346	0.331 ± 0.109	0.164	0.685	32.93	0.283	0.327	0.321 ± 0.115	0.155	0.692	35.82
	SPUE	−P	1.019	2.141	1.096 ± 0.327	0.569	1.762	29.83	0.909	1.639	1.175 ± 0.447	0.530	2.426	38.04
		+P	0.657	1.220	0.938 ± 0.303	0.327	1.630	32.30	0.768	1.000	0.913 ± 0.323	0.373	1.892	35.37
	PUE	−P	0.636	0.854	0.559 ± 0.205	0.270	1.155	36.67	0.572	0.826	0.537 ± 0.230	0.242	1.176	42.83
		+P	0.371	0.502	0.495 ± 0.181	0.209	0.936	36.56	0.409	0.462	0.474 ± 0.180	0.203	0.974	37.97
Yield trait	GY	−P	4.045	3.195	4.371 ± 1.327	2.013	7.913	30.35	3.721	2.939	4.140 ± 1.237	1.982	7.280	29.87
		+P	4.831	3.940	5.086 ± 2.861	0.515	16.607	56.25	5.459	4.452	5.521 ± 2.926	1.140	16.766	52.99
	SY	−P	3.680	2.037	3.933 ± 1.197	1.378	6.663	30.43	3.533	2.956	3.929 ± 1.215	2.006	6.397	30.92
		+P	4.659	3.026	4.544 ± 1.832	1.708	9.529	40.31	5.218	3.389	5.084 ± 2.079	1.913	10.672	40.89
	DM	−P	7.725	5.232	8.305 ± 2.384	4.277	13.087	28.70	7.254	5.895	8.070 ± 2.284	4.566	12.268	28.30
		+P	9.490	6.966	9.651 ± 3.629	3.437	20.228	37.60	10.677	7.841	10.603 ± 4.008	3.862	20.742	37.80

*PAE, P acquisition efficiency; PUE; for P use efficiency; GPC, Grain P concentration; SPC, Straw P concentration; PC, Plant P concentration; GPUP, Grain P uptake; SPUP, Straw P uptake; PUP, Plant P uptake; GPUE, Grain P utilization efficiency; SPUE, Straw P utilization efficiency; PUE, Plant P utilization efficiency; GY, Grain yield; SY, Straw yield; DM, Dry matter.*

Compared with those at −P, higher values were obtained for grain P concentration (GPC), straw P concentration (SPC), grain P uptake (GPUP), straw P uptake (SPUP) and plant P uptake (PUP) at +P. However, the values for grain P utilization efficiency (GPUE), straw P utilization efficiency (SPUG), and plant P utilization efficiency (PPUE) were higher at −P (Table 3). There were significant differences in these traits between the two parents under the two different treatments. Compared with the wild barley genotype CN4027, Baudin showed higher values for GPC, SPC, GPUP, SPUP, and PUP but lower values for GPUE, SPUG, and PUE (Table 3). The *CV* of the seven PAE- and PUE-related traits in the population between the two treatments also showed a wide distribution ranging from 31.80 to 62.54%. The transgressive segregation and approximately normal distributions could be also detected for the P-related traits (Table 3).

### Correlations Between P-Related and Yield Traits

Phenotypic correlations between P-related and yield traits under the two treatments were presented in Table 4. Significant correlations were detected between each of the three yield traits and most of the P-related traits under both P conditions (*P* < 0.01 or 0.05). P-concentrations related traits, including GPC, SPC, and PC, were significantly and negatively correlated with the three yield traits (GY, SY, and DM) under the −P treatment except SPC, PC in trial 2. P-uptake related traits, including GPUP, SPUP and PUP, were significantly and positively correlated with the three yield-related traits (GY, SY, and DM) except GPUP in trial 1 and GPUP and PUP in trial 2 (Table 4). Traits associated with PUE, including SPUE and PUE, showed a significantly positive correlation with two of the yield-related traits (SY and DM) with coefficients ranging from 0.205 to 0.508 (*P* < 0.05). PC was significantly and negatively correlated with GY and SY under +P treatment. Traits related with P-uptake, including GPUP, SPUP, and PUP, were significantly and positively correlated with DM in both trials with coefficients ranging from 0.362 to 0.748 (*P* < 0.01).

**TABLE 4:** Correlations between P- and yield-related traits in the RIL population at maturity in barley.

**Trial**	**Traits**	**Treatments**	**GPC**	**SPC**	**PC**	**GPUP**	**SPUP**	**PUP**	**GPUE**	**SPUE**	**PUE**
T1	GY	−P	−0.218*	−0.432**	−0.186*	0.528**	0.241**	0.523**	0.105	0.427**	0.150
		+P	0.034	0.046	0.401**	0.882**	–0.077	0.839**	–0.032	–0.040	−0.436**
	SY	−P	−0.309**	−0.513**	−0.423**	0.172	0.527**	0.253**	0.149	0.494**	0.337**
		+P	−0.246**	–0.112	−0.436**	−0.193*	0.783**	–0.024	0.187*	0.037	0.421**
	DM	−P	−0.288**	−0.520**	−0.330**	0.397**	0.417**	0.437**	0.139	0.508**	0.264**
		+P	–0.105	–0.020	0.123	0.693**	0.362**	0.748**	0.074	–0.016	–0.162
T2	GY	−P	−0.195**	–0.134	–0.090	0.511**	0.296**	0.559**	0.079	0.148	0.069
		+P	0.001	0.069	0.394**	0.857**	–0.034	0.826**	0.017	–0.063	−0.378**
	SY	−P	−0.354**	−0.202*	−0.449**	0.032	0.617**	0.618	0.268**	0.213*	0.391**
		+P	−0.250**	–0.181	−0.493**	–0.178	0.710**	0.016	0.170	0.173	0.510**
	DM	−P	−0.313**	−0.191*	−0.307**	0.310**	0.519**	0.414**	0.197*	0.205*	0.262**
		+P	–0.149	–0.048	0.047	0.639**	0.396**	0.709**	0.117	0.049	–0.024

PAE, P acquisition efficiency; PUE, for P use efficiency; GPC, Grain P concentration; SPC, Straw P concentration; PC, Plant P concentration; GPUP, Grain P uptake; SPUP, Straw P uptake; PUP, Plant P uptake; GPUE, Grain P utilization efficiency; SPUE, Straw P utilization efficiency; PUE, Plant P utilization efficiency; GY, Grain yield; SY, Straw yield; DM, Dry matter; *Significant at *P* ≤ 0.05 level. **Significant at *P* ≤ 0.01 level.

### Detection of QTL

**FIGURE 1:**
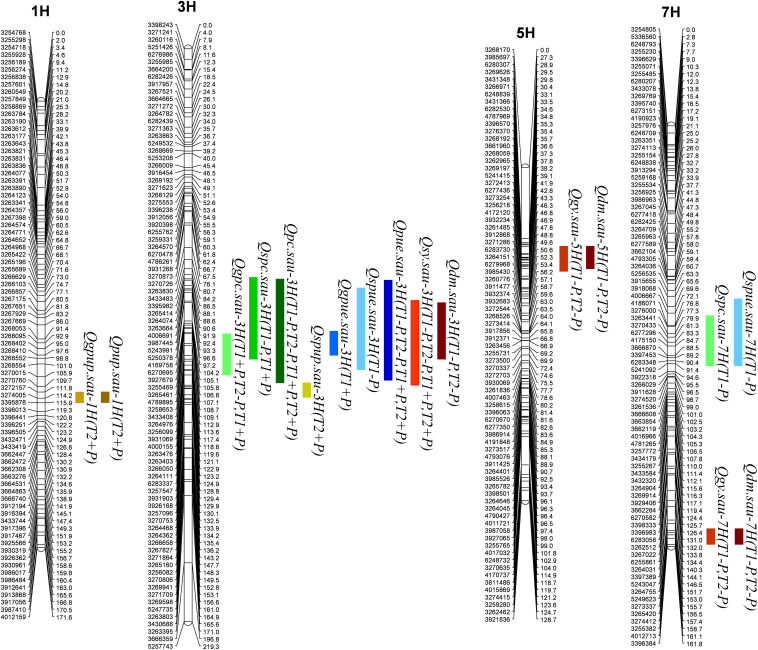
QTL for P- and yield-related traits detected in the RIL population.

A total of 17 QTL for P- and yield-related traits were identified. Phenotypic variations explained by these loci varied from 11.0 to 24.7% (Table 5 and Figure 1). LOD values for these loci ranged from 3.03 to 7.31 (Table 5). The 17 QTL were distributed on 4 chromosomes, including 1H (2 QTL), 3H (9 QTL), 5H (2 QTL), and 7H (4 QTL). Positive alleles for eight of these QTL were contributed by Baudin, while the remaining nine were contributed by CN4027. In addition, nine of these 17 QTL were detected in two trials, and three of them were detected at the two P conditions.

**TABLE 5:** QTL for P- and yield traits at maturity in barley.

**Traits**	**QTL**	**Ch.^a^**	**Detection environment**	**Marker interval**	**LOD**	**PVE(%)^b^**	**Additive^c^**
GPC	*Qgpc.sau-3H*	3H	T1−P	3264976–6283337	4.32	14.7	0.338
		3H	T2−P	3264976–3931069	3.90	13.4	0.380
		3H	T1+P	3433408–3264976	3.98	13.6	0.302
SPC	*Qspc.sau-3H*	3H	T1−P	4169758–4000155	5.01	17.9	0.100
		3H	T1+P	3264074–6248874	3.17	11.0	0.111
	*Qspc.sau-7H*	7H	T1−P	4186071–5241092	4.23	14.4	0.090
PC	*Qpc.sau-3H*	3H	T1−P	3264976–6283337	5.22	17.5	0.236
		3H	T2−P	3265461–4000155	5.98	21.0	0.272
		3H	T1+P	3264074–3264111	5.92	20.6	0.311
		3H	T2+P	3433483–4000155	4.67	17.0	0.277
GPUP	*Qgpup.sau-1H*	1H	T2+P	3272157–3395878	3.19	11.4	3.638
SPUP	*Qspup.sau-3H*	3H	T2+P	3257547–3926168	3.11	10.9	−0.811
PUP	*Qpup.sau-1H*	1H	T2+P	3272157–3395878	3.03	11.0	3.683
GPUE	*Qgpue.sau-3H*	3H	T1+P	3258653–3931069	4.82	16.5	−0.033
SPUE	*Qspue.sau-3H*	3H	T1−P	4006691–3266050	5.59	20.0	−0.120
	*Qspue.sau-7H*	7H	T1−P	3918068–5241092	3.98	13.6	−0.099
PUE	*Qpue.sau-3H*	3H	T1−P	3264976–3264111	3.68	12.7	−0.063
		3H	T2−P	3264976–3256099	4.22	15.3	−0.068
		3H	T1+P	3264074–6283337	7.31	24.7	−0.072
		3H	T2+P	3258653–3264111	4.88	17.9	−0.056
GY	*Qgy.sau-5H*	5H	T1−P	3266971–5241415	3.81	13.1	0.374
		5H	T2−P	3266971–3276370	3.54	12.6	0.338
	*Qgy.sau-7H*	7H	T1−P	3273337–4012713	3.55	12.3	−0.364
		7H	T2−P	3273337–3255382	3.28	11.4	−0.324
SY	*Qsy.sau-3H*	3H	T1−P	3264976–3263403	4.59	16.0	−0.378
		3H	T2−P	3264976–3263403	4.65	16.6	−0.390
		3H	T1+P	5250378–3257547	4.69	16.1	−0.651
		3H	T2+P	3433408–3257547	4.66	15.8	−0.735
DM	*Qdm.sau-3H*	3H	T1−P	4169758–4000155	3.95	14.3	−0.677
		3H	T2−P	3264976–4000155	3.64	13.7	−0.623
	*Qdm.sau-5H*	5H	T1−P	3266971–5241415	3.57	12.4	0.629
		5H	T2−P	3266971–5241415	3.74	13.0	0.608
	*Qdm.sau-7H*	7H	T1−P	3273337–4012713	3.57	12.6	−0.643
		7H	T2−P	3265420-3255382	3.41	11.8	−0.585

*^*a*^Chromosome. ^*b*^Percentage of the phenotypic variation explained by the QTL. ^*c*^Additive effect of a QTL. Positive values of additive effect indicate that alleles from Baudin are increasing the trait scores, and negative values indicate that alleles from CN4027 are increasing the score.*

One QTL (*Qgpc.sau-3H*) for GPC was detected on 3H, and its positive allele was derived from Baudin (Figure 1 and Table 5). *Qgpc.sau-3H* was detected under the two different P conditions in Trial 1 but only under −P condition in Trial 2. Phenotypic variations explained by this locus varied from 13.4 – 14.7 %. Two QTL for SPC were detected and they were mapped on chromosomes 2H and 7H, respectively. The positive alleles for both loci were contributed by Baudin (Figure 1 and Table 5). *Qspc.sau-3H* was detected at both +P and −P conditions in Trial 1, explaining 17.9 and 11.0% of the phenotypic variation, respectively. *Qspc.sau-7H* was detected at −P condition in Trial 1 and it explained 14.4% of the phenotypic variation. One significant QTL (*Qpc.sau-3H*) for PC was detected on chromosome 3H (Figure 1 and Table 5) under both P conditions in both trials conducted. The phenotypic variation explained by these QTL ranged from 17.0 to 21.0% (Table 5). The positive allele of *Qpc.sau-3H* was contributed by Baudin.

One QTL (*Qgpup.sau-1H*) for GPUP was detected on 1H under the +P condition from Trial 2, and its positive allele was derived from Baudin (Figure 1 and Table 5). This locus explained 11.4% of the phenotypic variation. One QTL (*Qspup.sau-3H*) for SPUP was detected on 3H, and the positive allele of it was derived from CN4027 (Figure 1 and Table 5). *Qspup.sau-3H* was detected at +P condition in Trial 1, and it explained 10.9% of the phenotypic variation. One QTL (*Qpup.sau-1H*) for PUP was detected on 1H under the +P condition from Trial 2, and its positive allele was also derived from Baudin (Figure 1 and Table 5). This locus explained 11.0% of the phenotypic variation.

One QTL (*Qgpue.sau-3H*) for GPUE was located on 3H under the +P condition from Trial 1 (Figure 1 and Table 5). This locus explained 16.5% of the phenotypic variation and its positive allele was derived from CN0427. Two QTL for SPUE were located, on 3H and 7H chromosome, respectively. Positive alleles for both QTL were derived from CN0427 (Figure 1 and Table 5). They explained 20.0 and 13.6% of the phenotypic variation, respectively. One stable QTL for PUE (*Qpue.sau-3H*) was detected on 3H under both P conditions from both Trial 1 and Trial 2 (Figure 1 and Table 5). It explained 12.7 – 24.7% of the phenotypic variation. The positive allele of this locus was derived from CN0427.

Three QTL (*Qdm.sau-3H*, *Qdm.sau-7H*, and *Qdm.sau-7H*) for DM were detected under the −P condition from the two trials. They were mapped on chromosomes 3H, 5H and 7H, respectively (Figure 1 and Table 5). The phenotypic variation explained by these QTL ranged from 11.8 to 14.3% (Table 5). The positive alleles of *Qdm.sau-3H* and *Qdm.sau-7H* were contributed by CN4027 and that of *Qdm.sau-5H* was contributed by Baudin. Two QTL (*Qgy.sau-5H* and *Qgy.sau-7H*) for GY were detected and they were mapped on chromosomes 5H and 7H, respectively (Figure 1 and Table 5). *Qgy.sau-5H* explained 12.6 and 13.1% of the phenotypic variation, respectively, and its positive alleles were contributed by Baudin. *Qgy.sau-7H* explained 11.4 and 12.3% of the phenotypic variation, respectively, and its positive allele was contributed by CN4027. A stable QTL (*Qsy.sau-3H*) for SY was detected on chromosome 3H under both the −P and +P conditions from both trials (Figure 1 and Table 5). It was derived from CN4027, and explained 15.8 – 16.6% of the phenotypic variation.

### Candidate Genes for the P-Related Loci

A total of fourteen candidate genes located in intervals harboring the P-related loci were detected by searching the BARLEX database. These candidate genes could be divided into four categories: acid phosphatase, phosphate transporter, acid phosphatase/vanadium-dependent haloperoxidase-related protein, and phospholipid metabolism (Table 6). The acid phosphatase gene (*AK354580*) and phosphate transporter gene (*MLOC_61737.2*) were identified in the intervals harboring *Qspue.sau-7H* and *Qspc.sau-7H* for PUE and PC. The candidate genes for the other two categories were located on three chromosomes and they confer PUE, PUP, and PC, respectively. One, one and two genes encoding acid phosphatase/vanadium-dependent haloperoxidase-related proteins were identified on 1H, 3H, and 7H, respectively. Two, two, and three genes associated with phospholipid metabolisms were identified on 1H, 3H, and 7H, respectively.

**TABLE 6:** Candidate genes or proteins in chromosomal intervals containing the various P-related loci at maturity in barley.

**Stable QTL**	**Chr**	**Gene Name**	**Functional Annotation**	***Oryza sativa***	***Zea mays***	***Arabidopsis thaliana***	**Functional Annotation**
*Qpup.sau-1H Qgpup.sau-1H*	1H	*MLOC_69370.3*	Acid phosphatase/vanadium-dependent haloperoxidase related	*LOC_Os05g47530.1*	*GRMZM2G177617_T05*	\	
		*MLOC_16149.3*	Digeranylgeranylglyceryl phosphate synthase	*LOC_Os07g38850.1*	*GRMZM2G113476_T03*	*AT3G11945.2*	Homogentisate prenyltransferase
		*AK356092*	Putative phosphatidylinositol transfer protein	*LOC_Os01g50616.1 LOC_Os05g46720.1 LOC_Os02g04020.1*	*GRMZM2G073571_T03 GRMZM2G171354_T01 GRMZM2G157043_T01 GRMZM2G174990_T03 GRMZM2G355610_T01*	*AT1G19650.1 AT1G75370.2 AT2G21520.2 AT4G39170.1*	Phosphatidylinositol/phosphatidylcholine transfer protein SFH4 Sec14p-like phosphatidylinositol transfer family protein Sec14p-like phosphatidylinositol transfer family protein Phosphatidylinositol/phosphatidylcholine transfer protein SFH4
*Qgpc.sau-3H Qspc.sau-3H Qpc.sau-3H Qspup.sau-3H Qgpue.sau-3H Qspue.sau-3H Qpue.sau-3H*	3H	*MLOC_56200.1*	Acid phosphatase/vanadium-dependent haloperoxidase related protein	*LOC_Os01g67560.1*	*GRMZM2G091435_T01*	\	
		*MLOC_53886.2*	2-phosphoglycerate kinase-related protein	*LOC_Os02g57400.1 LOC_Os09g39870.1*	*GRMZM2G017334_T01 GRMZM2G342327_T03 GRMZM2G123544_T01*	*AT5G60760.1 AT3G45090.1*	P-loop NTPase domain-containing protein LPA1 homolog 1
		*AK356601*	Phosphatidylinositol transfer protein SFH5	*LOC_Os05g35460.1 LOC_Os01g65380.1*	*GRMZM2G033641_T01 GRMZM2G081652_T01 GRMZM2G033649_T01*	*AT4G09160.1 AT1G72160.1*	Patellin-5 Patellin-3
		*MLOC_19234.6*	Phosphatidylinositol-4- phosphate 5-kinase	*LOC_Os12g13440.1 LOC_Os09g10650.1 LOC_Os08g01390.1 LOC_Os04g59540.1*	*GRMZM2G343218_T01 GRMZM2G428386_T02 GRMZM2G059179_T01 GRMZM2G040296_T01*	*AT1G34260.1*	Putative 1-phosphatidylinositol- 3-phosphate 5-kinase FAB1D
*Qspue.sau-7H Qspc.sau-7H*	7H	*AK354580*	Acid phosphatase 1	*LOC_Os06g36400.1*	*GRMZM2G103526_T01*	*AT4G29260.1 AT4G29270.1*	Acid phosphatase-like protein Acid phosphatase-like protein
		*MLOC_69490.1*	Acid phosphatase/vanadium-dependent haloperoxidase related protein	*LOC_Os08g03370.1*	*GRMZM2G057258_T01*	*AT1G24350.3 AT1G67600.1*	Acid phosphatase/vanadium-dependent haloperoxidase-related protein
		*MLOC_38965.4*	Acid phosphatase/vanadium-dependent haloperoxidase-related protein	*LOC_Os06g33930.1*	*GRMZM2G071638_T01*	*AT3G12685.1*	Acid phosphatase/vanadium-dependent haloperoxidase-related protein
		*MLOC_61737.2*	Phosphate transporter 1;8	*LOC_Os06g21950.1*	*\*	*AT1G20860.1 AT1G76430.1*	phosphate transporter 1;8 Putative phosphate transporter
		*AK362615*	Phospholipase DDHD1	*LOC_Os08g01920.1*	*GRMZM2G023335_T01 GRMZM2G318860_T02*	*AT1G31480.1*	Phospholipase SGR2
		*MLOC_22194.1*	1-phosphatidylinositol- 3-phosphate 5-kinase	*LOC_Os04g59540.1 LOC_Os08g01390.1 LOC_Os09g10650.1 LOC_Os12g13440.1*	*GRMZM2G040296_T01 GRMZM2G059179_T01 GRMZM2G428386_T02 GRMZM2G343218_T01*	*AT1G34260.1*	Putative 1-phosphatidylinositol- 3-phosphate 5-kinase FAB1D
		*AK367170*	1-phosphatidylinositol- 3-phosphate 5-kinase	*LOC_Os03g28140.1 LOC_Os06g14750.1 LOC_Os08g34950.1 LOC_Os09g23740.1 LOC_Os08g33200.1*	*GRMZM2G066876_T01 GRMZM2G092595_T01 GRMZM2G111208_T01 GRMZM2G132373_T01 GRMZM2G153722_T01*	*AT1G71010.1 AT3G14270.1 AT4G33240.1*	Putative 1-phosphatidylinositol- 3-phosphate 5-kinase FAB1C 1-phosphatidylinositol-3-phosphate 5-kinase FAB1B 1-phosphatidylinositol- 3-phosphate 5-kinase FAB1A

## Discussion

P is one of the macroelements for plants, and it was non-substitutable in many physiological and biochemical metabolisms. Plant production could be reduced or even fail completely when soil P is deficient. As most of the applied P cannot be absorbed by plants, improving P uptake and use could offer a better sustainable method than only relying on fertilizer application (Gong et al., 2016). To explore desirable genes for P efficiency in barley, we investigated several P-related traits based on a RIL population derived from a cross between the wild barley CN4027 and the barley cultivar Baudin under −P and +P conditions. A total of 17 QTL, forming five clusters, were detected on chromosomes 1H, 3H, 5H, and 7H under the two different P conditions. Two of the QTL clusters, located on 1H (for GPUP/PUP) and on 7H (for SPUE/SPC), respectively, are novel as no other QTL conferring P-relative traits has ever been reported on these chromosomes.

### How PAE and PUE Affect P Efficiency in Barley

The two parents of the mapping population used in this study showed relatively large differences in each of the investigated traits at maturity under both P conditions studied. The wild barley genotype CN4027 showed higher P utilization efficiency (GPUE, SPUE, PUE), while Baudin showed higher values in P acquisition efficiency traits (including GPC, SPC, PC, GPUP, SPUP, and PUP). The results from the phenotypic analysis were consistent with those from QTL identification in this study. QTL mapping revealed that positive alleles for most of the loci of PAE were derived from Baudin, indicating that this genotype had higher P acquisition efficiency than that of CN4027. Furthermore, positive alleles for QTL conferring GPUE and PUE were contributed by CN4027, implying that this genotype showed greater P utilization efficiency than that of Baudin.

The yield traits including GY, SY, and DM were significantly and positively correlated with PAE (GPUP, SPUP, and PUP) and PUE (GPUE, SPUE, and PUE) at both the −P and +P conditions (Table 4). This finding was highly consistent with those obtained at seeding stage in this population (Guo et al., 2017). And a similar result was observed in wheat (Su et al., 2009) and *Brassica napus* (Yang et al., 2011). While P concentrations including GPC, SPC, and PC were significantly and negatively correlated with most of three yield traits (Table 4). Thus, we think it will be challengeable to develop a cultivar with improved both PAE (PC and PUP) and PUE.

### The QTL for PAE and PUE

In this study, a total of 17 QTL for PAE, PUE, and yield traits were detected on five QTL clusters under two P conditions. A novel QTL cluster for SPUE/SPC was located on 7H under −P condition. Various candidate genes located in this QTL cluster were detected using database BARLEX searching as described in sorghum (Mahmoud et al., 2018). The acid phosphatase (AK354580) and phosphate transporter (MLOC_61737.2) genes located in this QTL cluster were identified in the interval of *Qspue.sau-7H* and *Qspc.sau-7H* (Table 6). The phosphate transporter 1;8 was a high affinity phosphate transporter which was reportedly a kind of phosphate transporter induced by low phosphorus (Raghothama, 2000). The acid phosphatase 1 was also induced by low phosphorus (Baldwin et al., 2001; Zhang et al., 2014). Thus, these two genes were likely important candidates for the QTL cluster for SPUE/SPC on 7H.

Three QTL clusters containing seven QTL for PAE and four QTL for PUE were identified on chromosomes 1H, 3H, and 7H. Candidate genes related to the acid phosphatase/vanadium-dependent haloperoxidase-related protein and phospholipid metabolism were located on these three QTL clusters. As an important phosphorus component in plants, phospholipid played a major role in phosphorus metabolic process. The expression of phospholipid metabolism genes was significantly different under the different P treatments (Pariascatanaka et al., 2009; Ren et al., 2011). However, the mechanisms of PAE and PUE regulated by phospholipid and acid phosphatase/vanadium-dependent haloperoxidase-related protein have not yet been reported, providing valuable clues for further dissecting their molecular mechanisms for P efficiency in barley.

It was reported that high P efficiency in plants could be achieved through improving PAE or PUE (Parentoni and Junior, 2008). Some scientists held the view that P efficiency was mainly determined by PAE (Parentoni and Junior, 2008; Richardson et al., 2009). While Veneklaas et al. (2012) hypothesized that PUE might play a major role in P efficiency. And it was reported that PUE and PAE were intrinsically linked (Su et al., 2006). The identified QTL clusters for several different traits might explain their significant correlations. For example, PAE and PUE showed significant correlation to three yield traits, and the QTL for these traits were all located in the same interval on 3H, indicating that they were linked closely or even be controlled by a same gene. Additionally, several QTL for PAE and PUE have been detected in the same region on 3H at seeding stage in barley (Guo et al., 2017). The QTL for PAE were also detected on 3A and 3B of bread wheat (Shi et al., 2008; Su et al., 2009). Chromosome 3H of barley was homologous to 3A, 3B and 3D of wheat (Islam and Shepherd, 1992), and the genes were highly conserved between wheat and barley (Devos, 2005). These results further verified the existence of a QTL cluster for P efficiency on 3H.

### The Challenge to Improve P Efficiency

An ideal P efficient genotype is usually characterized by high capacity to acquire more P in the P-deficient environment (i.e., PAE) and/or by high ratio of biomass and P content (i.e., PUE) (Guo et al., 2012). Results from the correlation analysis and QTL mapping indicated that enhancing PAE (including PUP, GPUP, and SPUP) and PUE would improve yield of barley under both +P and −P conditions. However, we observed that GPC and SPC would reduce yield at both +P and −P conditions. This means that it is not easy to simultaneously improve PAE and PUE. This finding is consistent with the results from Su et al. (2009) who reported that PAE and PUE were negatively correlated in wheat. A large number of QTL for P-efficiency have been reported in the last decade. However, few researches were utilized in crop breeding. We thus need to accelerate identifying major and stable QTL for PAE or PUE and developing their linked markers for MAS in barley breeding.

## Data Availability Statement

The raw data supporting the conclusions of this article will be made available by the authors, without undue reservation.

## Author Contributions

SG, JX, and SY developed the RIL population. SG, JX, YS, XZ, and GC performed the pot trials and tested yield traits. YuL, DH, JZ, TL, and YaL determined the P content of plant materials and soils. SG, JX, SY, and GC analyzed the data. SG, JX, and GC wrote the manuscript. GC conceived and designed the experiments. All the authors read and approved the final manuscript.

## Conflict of Interest

The authors declare that the research was conducted in the absence of any commercial or financial relationships that could be construed as a potential conflict of interest.

## Footnotes

1 http://wheat.pw.usda.gov/ggpages/wgc/98/Intro.htm

2 https://www.diversityarrays.com/

3 http://plants.ensembl.org/index.html

4 https://apex.ipk-gatersleben.de/apex/f?p=284:10

5 https://www.helmholtz-muenchen.de/pgsb
